# Rat cytomegalovirus efficiently replicates in dendritic cells and induces changes in their transcriptional profile

**DOI:** 10.3389/fimmu.2023.1192057

**Published:** 2023-11-23

**Authors:** Julia Cecilia Madela-Mönchinger, Silver Anthony Wolf, Emanuel Wyler, Agnieszka Bauer, Marius Mischke, Lars Möller, Vanda Juranić Lisnić, Markus Landthaler, Anna Malyshkina, Sebastian Voigt

**Affiliations:** ^1^ Department of Infectious Diseases, Robert Koch Institute, Berlin, Germany; ^2^ Genome Competence Center, Department of MFI, Robert Koch Institute, Berlin, Germany; ^3^ Laboratory for RNA Biology, Berlin Institute for Medical Systems Biology (BIMSB), Max Delbrück Center for Molecular Medicine in the Helmholtz Association (MDC), Berlin, Germany; ^4^ Institute for Virology, University Hospital Essen, University of Duisburg-Essen, Essen, Germany; ^5^ Advanced Light and Electron Microscopy, Robert Koch Institute, Berlin, Germany; ^6^ Center for Proteomics, University of Rijeka, Faculty of Medicine, Rijeka, Croatia

**Keywords:** cytomegalovirus, dendritic cells, transcriptome, chemokines, XCL1, XCR1

## Abstract

Dendritic cells (DC) play a crucial role in generating and maintaining antiviral immunity. While DC are implicated in the antiviral defense by inducing T cell responses, they can also become infected by Cytomegalovirus (CMV). CMV is not only highly species-specific but also specialized in evading immune protection, and this specialization is in part due to characteristic genes encoded by a given virus. Here, we investigated whether rat CMV can infect XCR1^+^ DC and if infection of DC alters expression of cell surface markers and migration behavior. We demonstrate that wild-type RCMV and a mutant virus lacking the γ-chemokine ligand *xcl1* (*Δvxcl1* RCMV*)* infect splenic rat DC *ex vivo* and identify viral assembly compartments. Replication-competent RCMV reduced XCR1 and MHCII surface expression. Further, gene expression of infected DC was analyzed by bulk RNA-sequencing (RNA-Seq). RCMV infection reverted a state of DC activation that was induced by DC cultivation. On the functional level, we observed impaired chemotactic activity of infected XCR1^+^ DC compared to mock-treated cells. We therefore speculate that as a result of RCMV infection, DC exhibit diminished XCR1 expression and are thereby blocked from the lymphocyte crosstalk.

## Introduction

During immunosuppression, opportunistic pathogens such as cytomegalovirus (CMV) contribute to increased morbidity and mortality. Due to its large genome bearing several immunomodulatory genes, CMV has the capacity to interfere with numerous immune cell types including dendritic cells (DC) (1–6).

As major antigen-presenting cells, DC link the innate and the adaptive immune response and play a central role in the activation of naïve T cells that are needed to neutralize invading pathogens (7). DC are crucial in the fight against viruses, and therefore, it is not surprising that they are targeted by different viral species. While conventional DC (cDC) can be infected with a multitude of viruses, plasmacytoid DC (pDC) resist most viral infections (8). Viruses targeting cDC include measles virus, influenza virus, HIV, hepatitis B and C viruses (9, 10), herpes simplex virus (11), CMV (12), and Varicella zoster virus (13), among others. Infection with measles virus abrogates the DC’s ability to stimulate the proliferation of naïve allogeneic CD4^+^ T cells (14). By contrast, influenza virus-infected DC were reported to induce a strong cytotoxic T cell response (15). Moreover, HIV exploits DC for dissemination to access other organs or cells (16, 17). Upon DC infection with murine CMV (MCMV), antigen uptake is impaired, DC phenotype is altered, and DC markers are downregulated (2). MCMV replicates productively in DC, recirculates with the help of M33, a virus-encoded chemokine receptor (18), and reaches the salivary gland via DC following intraperitoneal infection (19, 20). Hence, MCMV can remain in and modulate DC to escape an immune response.

While antigen presentation by MHC class I (MHCI) molecules to CD8^+^ T cells reports intracellular events, presentation of antigen adopted from the extracellular milieu occurs by MHC class II (MHCII) to CD4^+^ T cells. In addition, DC are able to present extracellularly acquired antigen by MHCI molecules to CD8^+^ T cells, a process known as antigen cross-presentation (21). In mice, DC capable of cross-presenting antigen are characterized by the expression of XCR1, a G protein-coupled receptor, which represents a lineage marker for this specialized DC subset (22). XCR1 is predominantly co-expressed on CD8a^+^ mouse cDC1 and CD141^+^ human cDC1 (23, 24). In rats, splenic, thymic, and lymph node-derived DC have been characterized (25–27). In particular, splenic DC could be separated into CD4^+^ and CD4^-^ subsets, and unlike CD4^-^ DC, CD4^+^ DC expressed CD5, CD90, and SIRPα/CD172a (28, 29).

In previous work, we showed that CD4^-^ XCR1^+^ DC, but not CD4^+^ XCR1^-^ DC, are attracted by supernatants of rat embryo fibroblasts (REF) infected with wild-type but not *Δvxcl1* rat CMV (RCMV (30)). So far, the only known γ-chemokine analogue *vxcl1* is encoded by the English (RCMV-E) and Berlin (RCMV-B) isolates of RCMV (31). vXCL1 has a sequence similarity of 63.2% (RCMV-E) and 65.5% (RCMV-B) to the amino acid sequence of endogenous rat XCL1, suggesting that it likely originated from a co-evolutionary adaptation process between the virus and the host (31). Since vXCL1 is exclusively attracting XCR1^+^ rat DC, we suggest that the virus manipulates this cell subset to evade the immune response.

Here, we extended previously reported rat DC analyses (27–29) to include XCR1 as an additional surface expression marker. By means of cross-presentation, XCR1^+^ DC can directly prime CD8 T cells, and this represents a promising approach for vaccine strategies against tumors (32). To better understand how RCMV manipulates DC and if these strategies can be extended into the field of antiviral approaches, we investigated whether or not RCMV is able to infect and replicate in isolated splenic DC. Finally, we analyzed DC phenotypes and determined transcriptional profiles by bulk RNA-sequencing.

## Materials and methods

### Preparation of single cell suspensions and enrichment of rat DC from spleens

Sprague Dawley rats were bred at the animal facility of the Robert Koch Institute and sacrificed by an isoflurane overdose. To obtain single cell suspensions, spleens were homogenized using a gentleMACS dissociator (Miltenyi Biotec). To increase DC yields, homogenates were digested with 500 µg/ml collagenase D, 20 µg/ml DNase I and 2% (v/v) FCS in RPMI 1640 for 25 min in a 37°C water bath shaking at 200 rpm. Digestion was halted by the addition of 10 mM EDTA for 5 min under the same conditions. Cell suspensions were filtered through a 100 µm nylon sieve (Becton Dickinson). After centrifugation at 380 x g for 8 min at 4°C, cells were applied to NycoPrep (PROGEN) or OptiPrep (PROGEN) density gradient centrifugation (density: 1.073 g/ml) at 1700 x g for 10 min at 4°C without applying de-acceleration. Lymphocytes were recovered from the NycoPrep/OptiPrep fractions and washed twice with MACS-PBS. To enrich OX-62-labeled cells, NycoPrep- or OptiPrep-enriched spleen cells were incubated with OX-62 microbeads (Miltenyi Biotec) according to the manufacturer’s instructions. To prevent unspecific Fc receptor binding, cells were incubated with rat gamma globulin (final concentration 45.2 µg/ml, Jackson ImmunoResearch) for 5 min on ice. Magnetically labeled cells were applied to a MS column (Miltenyi Biotec) for positive selection.

### Viruses and infections

Generation of the mutant *Δvxcl1* RCMV is described in (30). Likewise, a recombinant RCMV carrying *egfp* adjacent to *E32* was created by homologous recombination in eukaryotic cells. Both recombinant viral genomes were completely sequenced to rule out adventitious mutations that might have occurred during the recombination procedure, and none of such mutations were detected.

OX-62-enriched splenic DC were infected *ex vivo* with the respective viruses in chemotaxis medium (1x RPMI 1640, 1% [w/v] BSA [low-endotoxin; Gemini Bio-products], 100 U/ml Penicillin, 0.1 mg/ml Streptomycin, 50 µM β-mercaptoethanol) at MOI 3. Virus was UV-inactivated at 1 J/cm^2^ in 300 µl on a 6-well plate. Following OX-62 isolation, DC were either directly analyzed (input control) without cultivation or mock-infected and cultivated for 24 h before analysis.

### Antibodies and flow cytometry

Monoclonal antibodies (mAb) against RCMV-E immediate early 1 (IE1) protein were generated at the Center for Proteomics (University of Rijeka). Coupling of mAb to Alexa647 was conducted using a PD-10 desalting column (GE HealthCare). To characterize splenocytes, the following mAb were used: CD45RA (OX-33), CD54 (1A29), CD86 (24F), signal regulatory protein (SIRP)α/CD172a (OX-41), CD4 (W3/25), XCR1 (ZET), CD11b/c (OX-42), CD103 (OX-62; all from BioLegend); MHCII (OX-6) and CD103 (OX-62; both from BD Pharmingen); CD3 (REA227) and CD8a (REA437; both from Miltenyi Biotec). Titration of mAb was conducted for optimal signal-to-noise-ratio. Unspecific Fc receptor binding was inhibited by preincubation of cells with rat gamma globulin. Standard staining with mAb was performed in PBS containing 2% (v/v) FCS and 0.1% (w/v) NaN3 for 20 min on ice. For intracellular IE1 staining, OX-62-enriched DC were stained with the LIVE/DEAD Fixable Orange Viability staining kit (Thermo Fisher Scientific) and fixed with 2% (w/v) PFA. Permeabilization was conducted with 0.5% saponin. Data were acquired on flow cytometers (Fortessa, Becton Dickinson and MACSQuant10, Miltenyi Biotec) and evaluated using FlowJo software v10.4.2 (Tree Star).

### Chemotaxis assays

Chemotaxis assays were conducted as described in (33). Briefly, 2×10^5^ DC were added to the upper chamber of a Transwell system. Each Transwell contained 100 ng/ml (1×10^−8^M) rXCL1 or no chemokine in the lower chamber. After 2.5 h of incubation, cells that had migrated to the lower chamber were analyzed by flow cytometry. CD3^+^ and CD45RA^+^ cells were excluded by gating and MHCII^+^ CD103^+^ CD4^-^ XCR1^+^ DC were examined for migration. Cells were counted over 5 min and the percentage of migrated cells was calculated by dividing the number of migrated cells by the number of input cells x 100.

### Transmission electron microscopy

RCMV-infected DC were fixed with 2.5% (w/v) glutaraldehyde in 50 mM HEPES, pH 7.2, harvested by scraping, pelleted at 2000 x g for 5 min at 4°C, and washed twice with HEPES. After washing, cells were block-embedded by mixing equal amounts of centrifuged cells and low-melting-agarose (3%). Agarose-embedded cells were cut into small pieces (<1 mm) and postfixed with osmium tetroxide (1% in double distilled H_2_O for 1 h), tannic acid (0.1% in 50 mM HEPES for 30 min) and uranyl acetate (2% in ddH_2_O for 2 h). Agarose-embedded cells were dehydrated in a gradient series of ethanol and propylene oxide and finally embedded in Epon resin. Ultrathin sections (60 – 70 nm) were cut on a Leica-Ultracut ultramicrotome, mounted on naked 300 mesh copper grids, and counterstained with uranyl acetate (2% in ddH_2_O for 20 min), followed by lead citrate (Reynolds’ solution for 3 min). Ultrathin sections were stabilized with a thin layer of carbon evaporation and examined using a JEM-2100 transmission electron microscope (JEOL) at 200 kV. Images were recorded using a Veleta CCD camera (EMSIS).

### Fluorescent microscopy

REF cells were infected with supernatants acquired from GFP-encoding RCMV-, GFP-encoding RCMV UV-, and mock-infected primary DC cultures and subjected to fluorescent microscopy five days later. Microscopic images were collected with a Leica Thunder Imager (Leica Microsystems UK Limited, Milton Keynes, UK).

### RNA isolation and sequencing

Cultured and infected (MOI 3, 24 hours post infection; hpi) DC were prepared in triplicates. RNA was extracted using Trizol (Thermo Fisher Scientific) and purified from the aqueous phase using the RNA Clean & Concentrator 25 kit (Zymo Research). For sequencing, poly(A) RNA was extracted from OX-62-enriched DC using the Dynabeads mRNA DIRECT Kit (Thermo Fisher Scientific). Sequencing libraries were prepared using the NEXTflex Directional RNA-Seq Kit dUTP-based (Bioo Scientific) and sequenced on an Illumina HiSeq 2000 device to generate single end reads of 50bp length. After initial quality control (QC), including trimming of the adapter sequence AGATCGGAAGAGCACACGT, approximately 25–30 million mappable reads were obtained per sample. Raw sequencing reads were aligned to version rn6 of the rat genome using hisat2 (34), and reads quantified against the version 93 Ensembl annotation using quasR (35). Differential expression analysis was then performed using edgeR (36). 

### Quantitative RT-PCR

RNA was extracted from OX-62-enriched DC using innuPREP RNA Mini kit 2.0 and DNA removed by DNase I digestion. Of note, RNA amounts and integrity could not be assessed due to limited material, except for input samples. For those, around 20 ng RNA were isolated with a RIN >7. Subsequently, RNA was reverse transcribed using SuperScript III (ThermoFisher) with random hexamer priming according to the manufacturer’s protocol. For detection, cDNA was amplified using the 2x SYBR green PCR master mix (ThermoFisher) with standard conditions. The following (in case of spliced genes, exon-spanning) forward and reverse primer were used: 5′-ACGTGACATGGACTCAGACT-3′ and 5′-ACAAAACCAGGCTGTTACCCA-3′ (Rattus norvegicus Xcr1, Genbank acc. no. NM_001106871.1); 5′-ACCTCAGCTACAGGACGGAC-3′ and 5′-ATAGTCTCATTCCACCCAGTGC-3′ (ifnb1, NM_019127.2); 5′-TACCCTCTGTGGTTTCCAGC-3′ and 5′-TCCTTTGGTTTCTTGACCACCT-3′ (cd40; NM_134360.1); 5′-GGGCTCCTCTGAATCGACTG-3′ and 5′-GGCTACACCCCAAGAGCTTC-3′ (cdc25a; D16236.1); 5′-TCCTATGCCTCACAGATCCCA-3′ and 5′-AGGGTGCTTATGCACGTCTG-3′ (ccl6; NM_001004202.3). The housekeeping gene peptidylprolyl isomerase A (Ppia; 5′-TCTGCACTGCCAAGACTGAG-3′ and 5′-GTCCACAGTCGGAGATGGTG-3′; NM_017101.1) was used to normalize the data using the ΔΔ Ct method (37).

## Results

### Two major DC populations in the spleen can be identified by CD103 and CD4 staining

**Figure 1:**
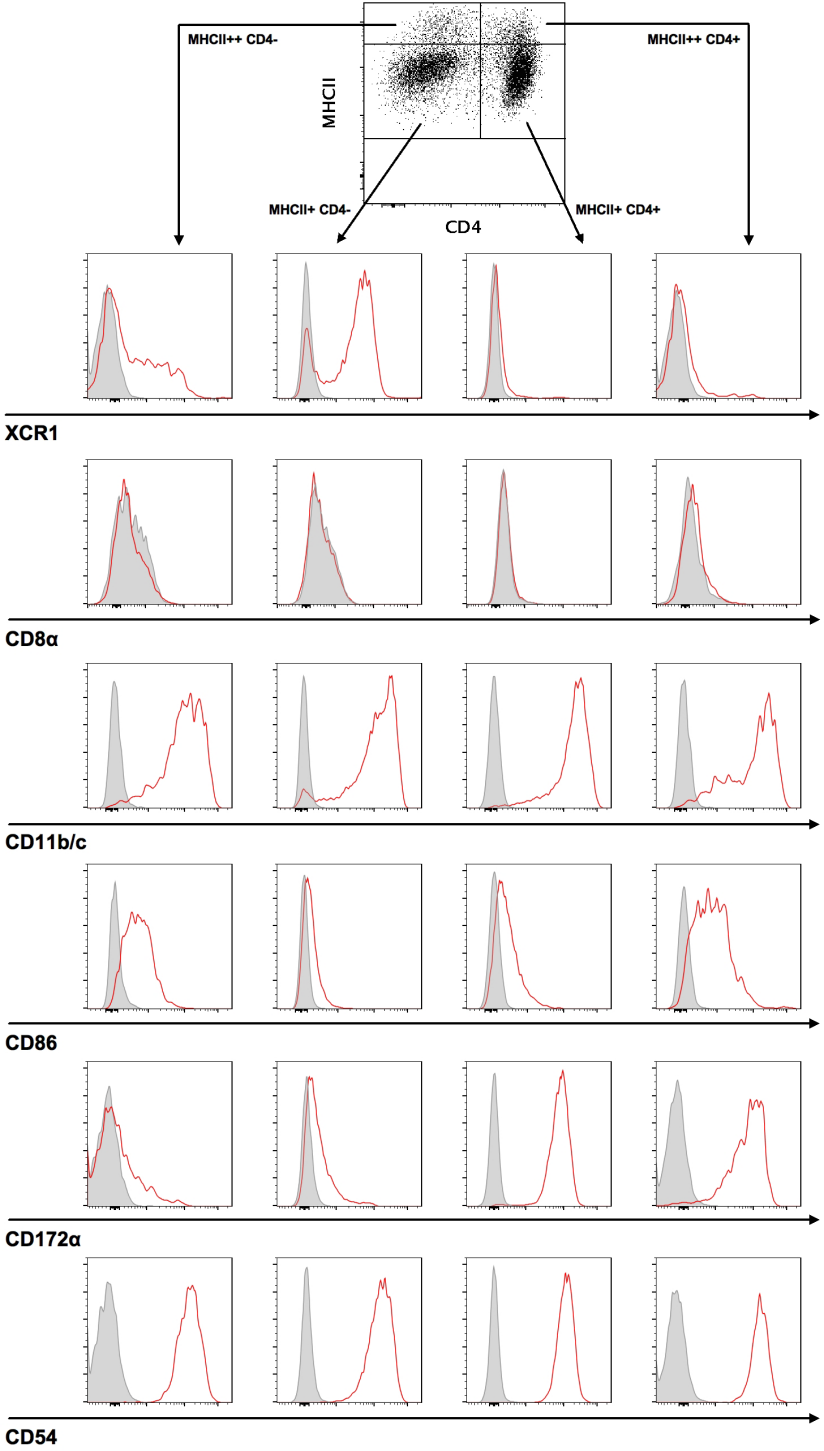
Two major DC subsets can be identified in splenocytes. For the characterization of DC subsets, splenocytes were stained with a target cell antibody panel and analyzed by flow cytometry. Cells were initially gated on singlets and live cells, followed by gating on CD3^-^ CD45RA^-^ CD103^+^ DC. Next, CD103^+^ DC were differentiated using MHCII and CD4 antibodies, divided into MHCII^++^ or MHCII^+^ and CD4^+^ or CD4^-^ DC, and subsequently characterized by antibody staining of XCR1, CD8α, CD11b/c, CD86, CD172α, and CD54. Representative histograms (red curve) for each analyzed surface marker are shown compared to a fluorescence minus one (FMO) control (grey curve).

In order to investigate the permissiveness of rat DC in RCMV infection, we initially defined a strategy to characterize DC subsets in spleens from Sprague Dawley rats. Splenocytes from ten-week-old rats were freshly isolated and enriched by OptiPrep density centrifugation and analyzed by flow cytometry. After isolating CD103^+^ DC and gating on this population, we used MHCII expression levels to identify four DC subsets: CD4^-^ MHCII^+^, CD4^-^ MHCII^++^, CD4^+^ MHCII^+^, and CD4^+^ MHCII^++^ DC. Based on CD4 expression, we further identified two major DC populations: MHCII^+/++^ CD103^+^ CD4^-^ and MHCII^+/++^ CD103^+^ CD4^+^ DC. These markers were then used to differentiate between two DC subpopulations: a) MHCII^+^ CD103^+^ CD11b/c^+^ CD4^+^ SIRPα/CD172a^+^ XCR1^-^; and b) MHCII^+^ CD103^+^ CD11b/c^+^ CD4^-^ SIRPα/CD172a^-^ XCR1^+^ (**Figure 1**). CD11b/c, CD54 and, to a lesser extent, CD86 could be detected on both DC subsets, while SIRPα/CD172a was strongly expressed on the CD4^+^ and dim on the CD4^-^ subset. In contrast to mouse DC, CD8 expression remained undetected on rat spleen-derived DC. XCR1 was almost exclusively present on CD4^-^ SIRPα/CD172a^-^ DC. In all cell preparations, the purity of CD4^-^ MHCII^+^ cells used for viability or chemotaxis assays was above 85%.

### RCMV infects and replicates in splenic DC *ex vivo*

**Figure 2:**
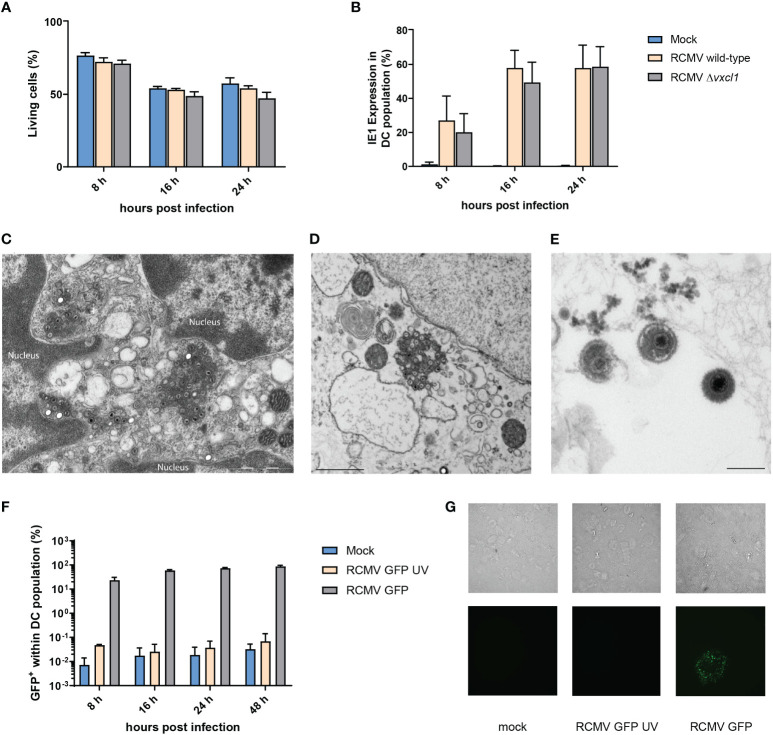
Viability of OX-62-enriched DC after infection with wild-type and *Δxcl1* RCMV and identification of replication compartments. DC were analyzed after 8, 16, and 24 hpi. **(A)** OX-62-enriched DC were infected with wild-type or *Δxcl1* RCMV. Mock-infected cells served as a negative control. **(B)** Expression of intracellular IE1 was analyzed by flow cytometry. Twenty-four hours post infection, ultrathin section transmission electron microscopy from OX-62-enriched DC revealed **(C)** viral capsids originating from cytoplasmic virus factories after nuclear egress and **(D, E)** mature viral particles outside the cell. **(F)** Recombinant GFP-encoding RCMV with or without UV inactivation. **(G)** Enriched DC were either mock-infected or infected with GFP-encoding RCMV with or without UV inactivation. Twenty-four hpi, supernatants were replaced with fresh culture medium. Forty-eight hours post initial infection, supernatants were collected and used to infect a REF monolayer at 75-80% confluency. Representative microscopic images of methylcellulose-overlayed REF cells are shown (bright-field upper row, GFP fluorescence lower row, 20x magnification).

Since DC play a central role in the initiation and regulation of immune responses during CMV infection, we investigated the permissiveness and cellular response of DC to RCMV infection. After infection of freshly OX-62-isolated splenic DC from 10-week-old rats with wild-type and *Δvxcl1* RCMV, we could not observe a pronounced impact on cell viability, as assessed by LIVE/DEAD Fixable Orange Viability staining (**Figure 2A**). After 8, 16, and 24 hpi, the percentage of viable cells remained consistently higher in mock- than in RCMV-infected cells, albeit no drastic difference was observed. However, cultivation of DC was limited to 48 h because viability decreased to 40% (data not shown). To document viral infection, we measured expression of RCMV IE1 protein at 8, 16 and 24 hpi. Eight hpi, infection of CD103^+^ DC was detected, and similar IE1 expression was seen after infection with both viruses 24 hpi (**Figure 2B**). As IE1 detection might not indicate replication-competent virus but merely an abortive infection, we assessed productive viral infection of DC by electron microscopy 24 hpi. We identified cytoplasmic virus factories after nuclear egress (**Figure 2C**) as well as mature particles outside the cell (**Figures 2D, E**). These data were also confirmed by using a recombinant RCMV carrying an *egfp*-cassette adjacent to *E32* and an UV-inactivated virus. As expected, infection with the *egfp*-containing virus resulted in a positive EGFP signal, while the UV-inactivated virus lacked GFP signal expression (**Figure 2F**). Moreover, wild-type RCMV-infected DC released infectious virus particles since supernatants collected from these DC infected REF cells (**Figure 2G**).

### RCMV infection affects DC phenotype and chemotactic activity

**Figure 3:**
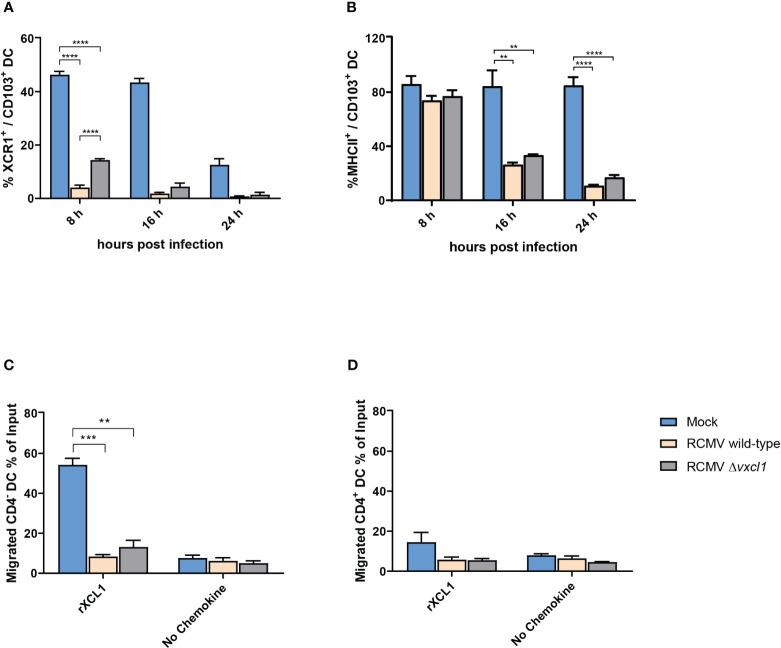
Analysis of DC surface markers and migration of CD4 DC after infection with RCMV. DC were analyzed after 8, 16, and 24 hpi. OX-62-enriched DC were infected with wild-type or *Δvxcl1* RCMV. Mock-infected cells served as a negative control. XCR1 **(A)** and MHCII **(B)** were stained. Migration of CD4^-^ **(C)** and CD4^+^ **(D)** DC were analyzed in the presence or absence of recombinant rat XCL1 (rXCL1). Error bars represent mean ± SD, n=3 independent experiments. ** p<0.001; *** p<0.0001; **** p<0.00001; (one-way ANOVA and Dunnett’s multiple comparison test).

Having shown that RCMV can productively infect DC, we investigated the impact on phenotype and motility changes. Both wild-type and *Δvxcl1* mutant virus infection led to a strong reduction in XCR1 surface expression (**Figure 3A**). In contrast to mock infection, RCMV wild-type and *Δvxcl1* mutant virus-infected DC showed significantly decreased XCR1 expression at 8 and 24 hpi. Further, we observed reduced MHCII expression at 16 hpi and 24 hpi (**Figure 3B**). To examine whether RCMV infection impairs functional properties, we tested the chemotactic activity of DC 4 hpi in a transwell assay in the presence or absence of 100 ng/ml recombinant rXCL1. While mock-infected CD4^-^ XCR1^+^ DC migrated toward recombinant chemokine, both wild-type and *Δvxcl1* RCMV-infected DC failed to migrate. Likewise, cell movement did not occur in the absence of recombinant rXCL1 (**Figure 3C**). As a control, infection of CD4^+^ DC did not exhibit chemotactic activity, irrespective of rXCL1 addition, as expected (**Figure 3D**).

### Cultivation and RCMV infection of DC induce changes in their transcriptional profile

As we could detect changes in the expression of selected DC surface molecules like XCR1 and MHCII by flow cytometry after RCMV infection, we extended our analyses to examining the whole transcriptome of RCMV-infected DC. As cultivation of isolated DC itself might induce transcriptomic changes, we initially compared the transcriptome profiles of freshly OX-62-isolated, uncultured, mock-infected CD4^-^ CD172α^-^ DC with mock-infected, 24 h cultured CD4^-^ CD172α^-^ DC.

**Figure 4:**
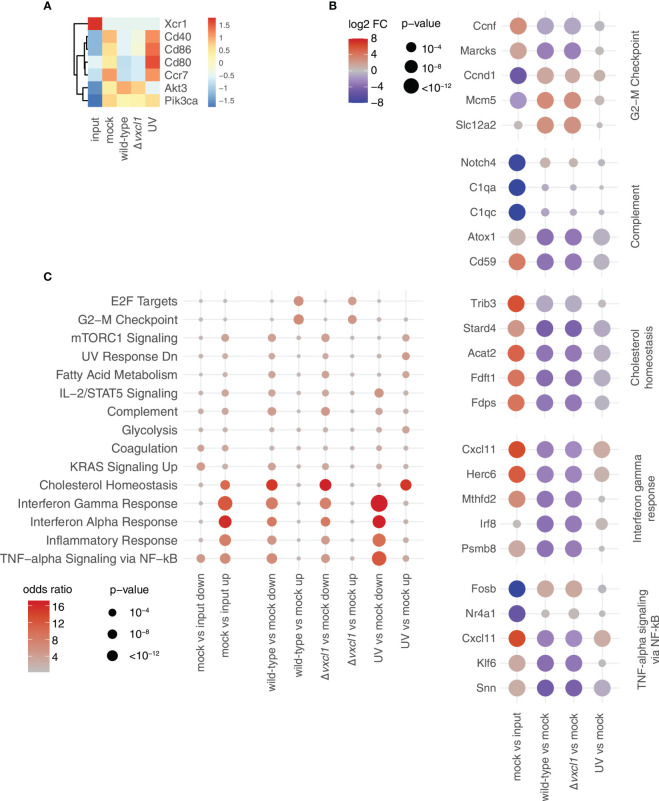
Transcriptomic changes upon DC cultivation and RCMV infection. **(A)** Expression values of the indicated DC markers for the five conditions analyzed are depicted as z-scores of normalized expression values (transcripts per million, tpm) averaged over three replicates from the RNA-seq data. **(B)** For the indicated comparisons, the top 500 up/downregulated genes, respectively, were used for gene set enrichment analysis using enrichR. For every gene set, the terms shown are the union of the five terms with the smallest adjusted p-values. Data are shown as dot plots with -log10 transformed adjusted p-value as the size, and the log2 transformed odds ratio (i.e., how many more genes are assigned to a term in the indicated gene set than expected) represented by the color. Rows are ordered according to an unsupervised clustering. For calculation of adjusted p-values, enrichR uses a hypergeometric test with FDR correction. **(C)** For selected terms, the five genes with the smallest p-values as calculated by the edgeR package used for differential expression analysis across all comparisons are indicated at the bottom. Values are shown as dotplots, with the size of the dot proportional to the -log10 transformed p-values, and the color indicative of the log2 transformed fold change. Within each term, genes are ordered according to an unsupervised clustering. For calculation of p-values, edgeR uses an exact two-sided binomial test.

Indeed, mere cultivation of rat splenic DC changed their transcriptomic profile, as compared with freshly isolated DC, suggesting that DC become activated during cultivation (**Figure 4A**, input vs. mock). Following cultivation, 2068 and 2505 genes out of about 11000 genes were significantly (p < 10^-6) and at least 2-fold up- or downregulated, respectively (i.e., log(2) transformed fold change larger than one/less than minus one; **Supplementary Table 1**). Upregulated genes with higher expression following cultivation (i.e., higher in “mock” compared to “input” condition) included signaling proteins related to DC migration such as *Pik3ca* and *Akt3* as well as maturation markers such as *Cd86*, *Cd80*, *Cd40*, and *Ccr7*. In contrast, XCR1 mRNA was barely detectable 24 h after incubation at 37°C in all sample groups (**Figure 4A**, **Supplementary Table 1**). This ligand-independent reduction of XCR1 mRNA confirmed the observed diminishment in surface expression, as shown above.

**Figure 5:**
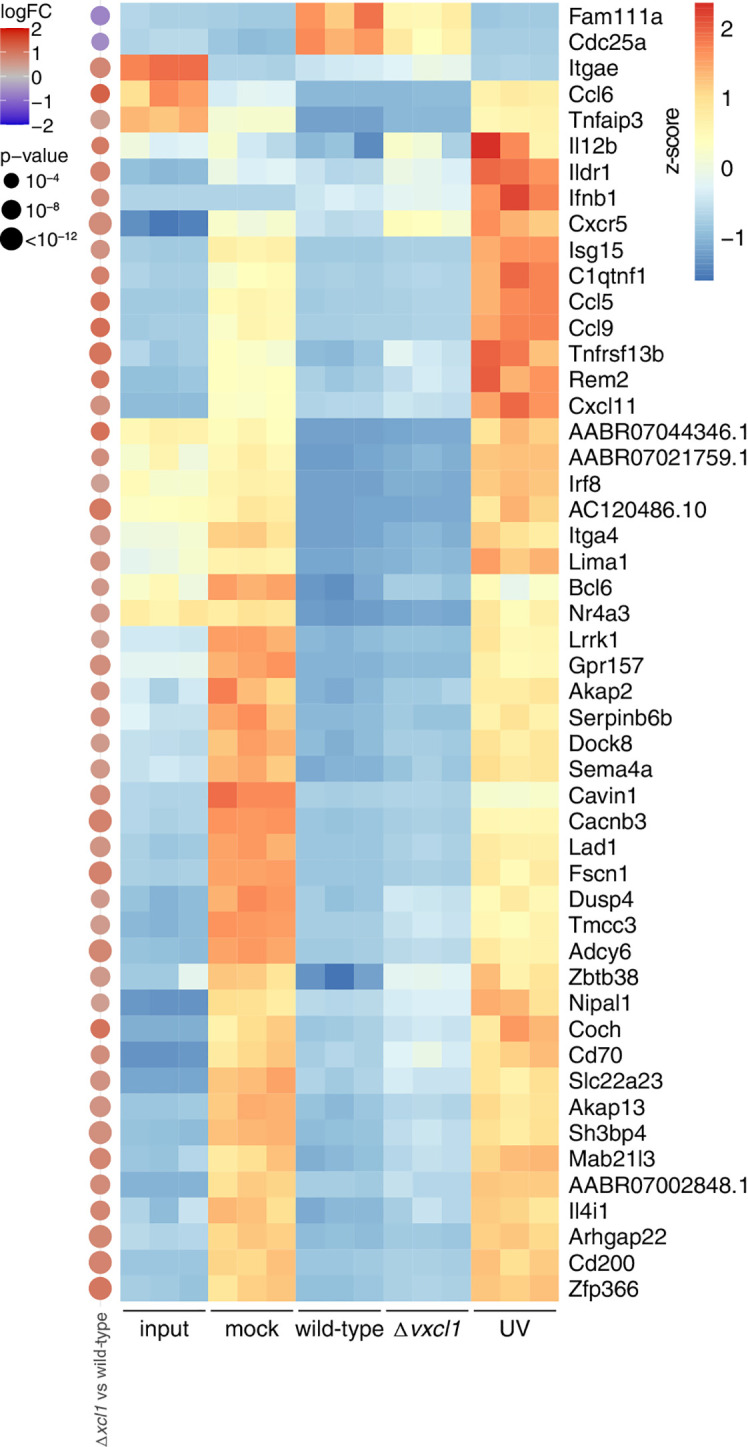
Transcriptomic changes upon infection with wild-type, *Δvxcl1*, and UV-inactivated RCMV. Expression values of the indicated differentially expressed genes (top 50 differentially expressed of *Δvxcl1* RCMV compared to wild-type RCMV infection) are depicted as z-scores of normalized expression values (tpm) of the individual samples (replicates 1-3) in the RNA-Seq data. On the left, differential expression values as calculated by edgeR for *Δvxcl1* RCMV vs. wild-type RCMV are shown, with the size of the dot proportional to the -log10 transformed p-values, and the color indicative of the log2 transformed fold change. For calculation of p-values, edgeR uses an exact two-sided binomial test.

Next, we performed an unbiased gene set enrichment analysis with the genes that show differential expression between the different conditions (**Figure 4B**). Among the terms with the strongest signal were interferon response, complement, as well as two terms related to cell cycle (“E2F targets” and “G2-M checkpoint”). We then investigated five of these terms more closely. For each of them, we selected the five genes with the strongest deregulation after analysis of all comparisons (e.g., mock vs. input, wild-type vs. mock etc; **Figure 4C**). First, we observed that genes that were upregulated in mock-infection vs. input were frequently downregulated in both wild-type and Δ*vxcl1* virus infections, such as cytokine-encoding *Cxcl11*, or *Fdft1* and *Fdps*, which are both coding for enzymes in the cholesterol synthesis pathway (**Figure 4B**). Vice versa, genes downregulated in comparing mock-infection vs. input, such as the MAP kinase target gene *Fosb*, were upregulated in infections compared vs. mock. For these genes, UV-inactivated virus-infected DC showed gene expression levels much closer to mock-infection. This “reversing pattern”, i.e., that changes in gene expression induced by the cultivation of DC were reverted by RCMV infection, was also observed for cell cycles genes (**Figure 4C**, top panel). In addition, we found that genes upregulated in mitosis such as *Top2a*, *Cenpe* or *Ube2c* exhibited stronger expression in infected DC, indicating that these genes contribute to cell division (**Supplementary Figure 1A**). Second, differences between mock- and UV-inactivated virus-infected DC were considerably smaller than those between non-inactivated virus and mock-infections. UV-inactivated virus is unable to replicate and cannot initiate large parts of its gene expression program. Accordingly, we detected substantial differences between wild-type and UV-irradiated RCMV infection (**Figure 5**). Differences in DC gene expression after wild-type and *Δvxcl1* RCMV infection were comparatively small, with only about 50 genes showing significant differences and reproducible expression patterns (**Supplementary Figure 1B**).

To validate our RNA-Seq results, we newly isolated DC from two rats and performed quantitative RT-PCR based on a set of genes that cover different terms including cell cycle and innate immunity pathways. Messenger RNA was extracted from OX-62-sorted DC that were either mock-infected, UV-infected, infected with wild-type, or *Δvxcl1* RCMV *in vitro*. Xcr1, Ifnb1, Cd40; Cdc25a, and Ccl6 mRNA were quantified and compared to input DC mRNA as a reference. The housekeeping gene peptidylprolyl isomerase A (Ppia) was used as a reference gene for normalization of quantitative RT-PCR data. The RT-PCR results could mostly recapitulate the data obtained with the RNA-Seq approach, although input values were generally higher in RT-PCR analyses (**Figure 5**, **Supplementary Figure 2**).

## Discussion

Initially, we performed a phenotypic characterization of rat DC and identified two main DC subsets in the spleen: MHCII^+^ CD103^+^ CD11b/c^+^ CD4^+^ SIRPα/CD172a^+^ XCR1^-^ DC on the one hand and MHCII^+^ CD103^+^ CD11b/c^+^ CD4^-^ SIRPα/CD172a^-^ XCR1^+^ DC on the other. Thus, our data confirm previous results from rat DC studies (25–29), including the observation that, in contrast to murine DC, splenic rat DC lack CD8a expression (38). We added the GPCR XCR1, the γ-chemokine receptor, to include a marker that defines cross-presenting DC in mice and humans (39, 40). In rats, cross-presentation data of XCR1^+^ DC are unpublished; phenotypically, XCR1 appears to be almost exclusively expressed on CD4^-^rat DC.

Next, we analyzed DC viability under different conditions. Overall, freshly isolated DC (input), compared with wild-type and *Δvxcl* RCMV-infected DC, did not exhibit major differences in viability at a given time point, but overall viability declined. Following MCMV infection, DC viability was also robust, however, a growth factor-dependent immature mouse DC line was used in that study (2, 41). Infection of DC with wild-type or *Δvxcl1* RCMV resulted in IE1 expression and in GFP expression after infection with a GFP-expressing recombinant virus. Here, we define DC infection not merely as the detection of viral IE1 or GFP expression as these could document successful entry but also an abortive infection; furthermore, infection should include the visualization of viral progeny. Successful viral replication could be confirmed by electron microscopy detection of capsids after nuclear egress and the identification of cytoplasmic virus factories. Moreover, primary DC cultures were productively infected since culture supernatants were infectious for REF.

RCMV is the only known virus to have evolved a γ-chemokine analogue, vXCL1, that engages its cognate receptor XCR1 and thereby attracts XCR1^+^ DC (30, 31, 33). Like other CMV, RCMV encodes a number of immune evasion genes that might have prompted the host to employ cross-presentation, and RCMV might use vXCL1 to circumvent this strategy of the immune system. After CMV infection of cells that have become apoptotic, DC cross-present ingested viral antigens to CD8^+^ T cells (42). This feature might be impaired upon direct infection of DC, however, the permissiveness of cross-presenting DC might serve as an antiviral strategy to prime CD8^+^ T cells (43). In addition to RCMV infection, DC function might be dampened by infected cells secreting vXCL1 that bind XCR1 and result in XCR1 downmodulation, thereby rendering this DC subset unresponsive. Also, vXCL1 might attract uninfected DC to facilitate viral dissemination, suggesting that vXCL1 (as XCL1) acts rather locally than over large distances.

Following infection with MCMV and the Maastricht isolate of RCMV, DC phenotypic changes including diminished MHCII expression have been reported (2, 44). Infection with RCMV-E induced a similar reduction in MHCII expression that became profound 16 hours and 24 hpi. Likewise, we detected reduced XCR1 surface expression after RCMV infection that was visible already at 8 hpi. At this time point, XCR1 expression was less pronounced after infection with wild-type RCMV which might indicate that *Δvxcl1* RCMV could be less successful in attracting and subsequently infecting DC. On the mRNA level, XCR1 was barely detectable after 24 h in all sample groups (data not shown), confirming our previous published results concerning the ligand-independent reduction of XCR1 expression (33). Both wild-type and *Δvxcl1* RCMV infection impeded migration of CD4^-^ DC toward endogenous XCL1, thereby possibly interfering with DC recruitment by NK cells and CD8^+^ T cells, as they constitute the main XCL1 source.

By comparing the transcriptomic profiles of uncultured DC with 24 h cultured DC, we sought to determine whether cultivation of isolated DC itself induced different patterns of gene expression. In our RNA-Seq analysis, we revealed major changes between DC that were either uncultured (“Input”) or mock-infected and cultured (“Mock”). Rat splenic DC became activated during cultivation, and maybe even during the isolation procedure including digestion, gradient centrifugation and microbead labeling, a phenomenon that has also been described for murine DC (2, 28, 45). Similarly, we observed decreased transcription of the maturation markers *Cd86*, *Cd80*, *Cd40*, and *Ccr7*, as after MCMV infection (2), suggesting that RCMV hampers DC maturation. *In vivo*, DC maturation is essential for the migration and initiation of T cell-derived immune responses. The inhibition of maturation might result in a paralyzed state and DC dysfunctionality, resulting in downstream T cell impairment, as has been described for herpes simplex virus, vaccinia virus and human CMV (46–48). In contrast, *Xcr1* was highly transcribed in uncultured cells. This transcriptional state flipped after 24 h of cultivation when *Xcr1* transcription became hardly detectable. Also, RCMV infection led to a rapid reduction of *Xcr1* transcription which is likely not only due to the infection process but also to XCR1 recycling (33).

After RCMV infection of cultured DC, 2001 and 1954 genes were significantly up- or downregulated, respectively, compared to mock-infection. We detected a substantial upregulation of genes involved in cell cycle and mitosis. However, upon DC cultivation, these genes were less abundantly expressed, which might suggest a G1 arrest in this condition that is reverted in infected cells. By contrast, several cytokines showed enhanced transcription only after mock-infection or infection with irradiated virus, possibly indicating the suppression of these genes upon viral encounter. In a published dataset, 130 murine and human DC genes were regulated upon maturation that contained putative transcription factor binding sites for IRF and NF-κB and therefore, maturation seems to be driven by genes controlled by IFN and NF-κB (49). In another study, transcriptomic changes were investigated in human myeloid DC after human CMV infection. Almost 200 genes were upregulated 6 hpi, and approximately 600 genes were upregulated and approximately 300 downregulated at 16 hpi (50).

To observe for changes in the DC transcriptomic profile, we compared wild-type virus to *Δvxcl1* RCMV infection. Overall, expression of DC genes after infection with wild-type and *Δvxcl1* RCMV were quite similar, yet there were about 50 genes that exhibited expression of significant difference. Among these were *Cxcr5*, *Il12b*, and *Cd200* that all play a role in antiviral immunity. As with rat DC, *Cd200* was also found to be upregulated in murine DC upon maturation (49). While both wild-type as well as *Δvxcl1* RCMV infection decrease *Cd200* transcription, lack of vXCL1 seems to allow for higher *Cd200* gene expression. Decreased CD200 expression on the DC surface might lead to DC tolerization and influence (v)XCL1 binding to XCR1, thereby impairing cross-presentation and subsequent T cell activation. Therefore, it can be speculated that RCMV employs vXCL1 to reduce CD200 abundance on DC.

Our RNA-seq data show that cultivation as well as RCMV infection of DC led to changes on the transcriptional level. We could detect similar changes in mRNA expression by quantitative RT-PCR for *Xcr1, Cdc25a*, and *Ccl6* and mainly for *Ifnb1* and *Cd40*. For *Ifnb1*, UV-treated RCMV showed highest expression as with RNA-seq, however, expression was detected in the mock-infected sample. For *Cd40*, mRNA expression was seen in the mock-infected but less in the UV-treated RCMV-infected sample. Generally, we detected high expression of input mRNA with RT-PCR that was not seen with RNA-seq. This might be due to the higher amount of mRNA detectable in the input sample as well as the difference of the two methods. While the results of the different infection conditions are comparable between the methods, this does not account for the input condition.

In summary, we show that maturation marker genes and genes regulating the cell cycle are affected by RCMV infection and identified different transcription profiles that depend on *vxcl1* transcription. Therefore, the analysis of the interaction between RCMV and XCR1^+^ DC should shed further light on CMV-induced immunosuppression and might be useful to improve antiviral or antitumor therapies by targeting cross-presenting DC.

## Data availability statement

The data presented in the study are deposited in the NCBI GEO repository, accession number GSE232184.

## Author contributions

JCM-M, SW, LM, AB, MM, AM, and EW performed experiments. VJ and ML provided reagents. JCM-M, SW, EW, and AM analyzed the data. JCM-M, EW, and SV wrote the manuscript. SV supervised the project. All authors contributed to the article and approved the submitted version.

## Supplementary material

Supplementary Figure 1
**(A)** Expression values of the indicated mitosis markers in the five conditions are depicted as z-scores of normalized expression values (tpm) averaged over three replicates from the RNA-Seq data. **(B)** Fold changes of the indicated differentially expressed genes (top 25 up and downregulated after RCMV infection compared to mock-infected samples) of the four conditions indicated at the bottom compared to mock are shown as dotplots, with the size of the dot proportional to the –log10 transformed p-values, and the color indicative of the log2 transformed fold change. The genes are ordered according to an unsupervised clustering.

Supplementary Figure 2 Quantitative RT-PCR of mRNA extracted from OX-62-enriched DC from a total of two rats. After isolation and enrichment, DC were mock- or UV-infected or infected with wild-type or *Δvxcl1* RCMV (see colored bars). RT-PCR was carried out to quantitate Ccl6, Cd40, Cdc25a, Ifnb1, and Xcr1 mRNA. Relative mRNA expression is shown on the ordinate. The housekeeping gene Ppia was used to normalize the data using the ΔΔ Ct method and input mRNA (red) was used as a reference to calculate ΔΔ Ct values. Averages and individual measurements of two rats each are shown. Error bars denote standard deviation.

Supplementary Table 1 Differential expression values of mock-infected compared to input cells.
