# Supplementary figures and images for: Rat cytomegalovirus efficiently replicates in dendritic cells and induces changes in their transcriptional profile
Source: Front Immunol. 2023 Nov 23;14:1192057. doi: 10.3389/fimmu.2023.1192057 (PMC10702230; doi:10.3389/fimmu.2023.1192057)

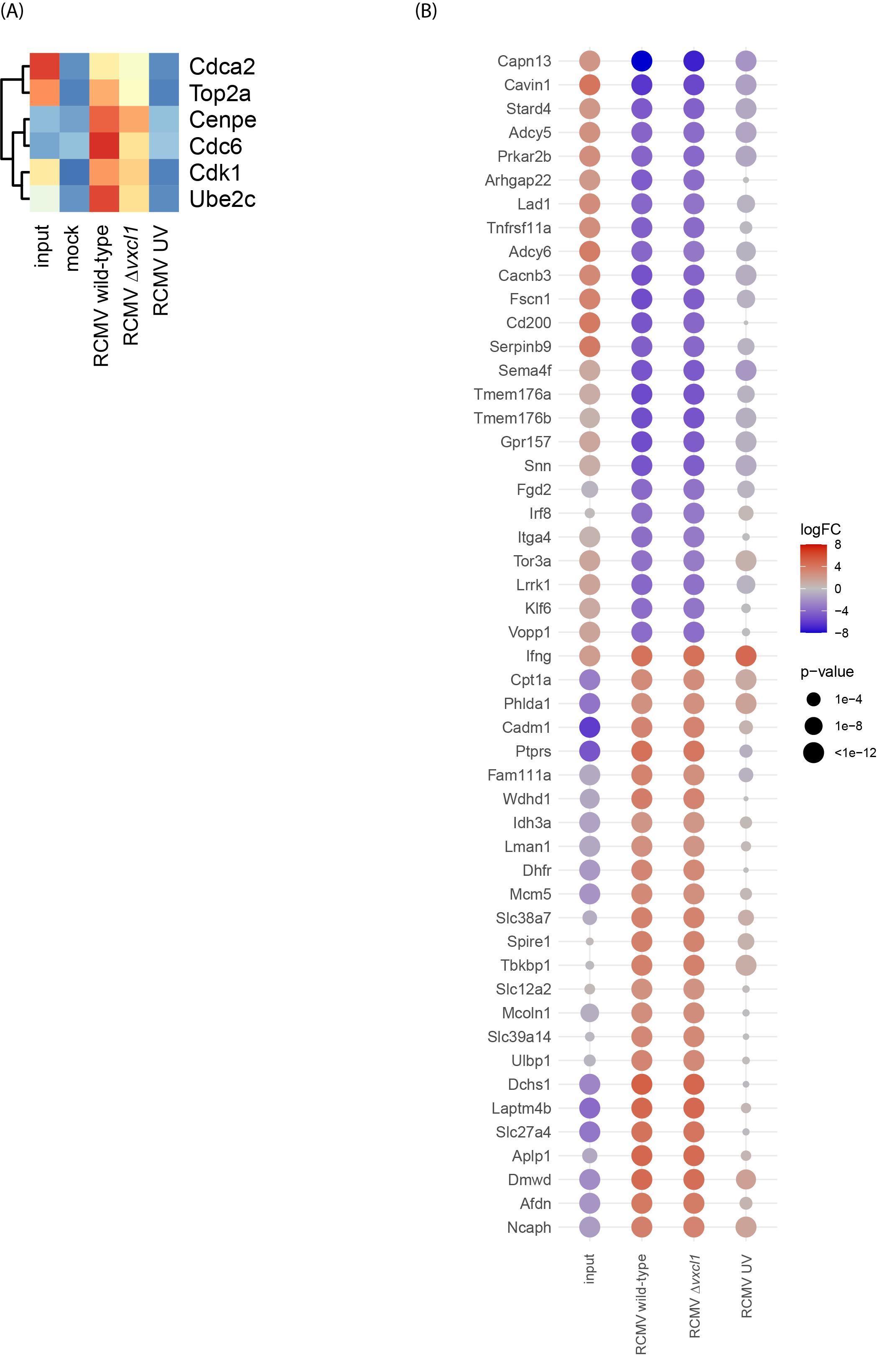

Supplement: Supplementary Figure 1 — (A) Expression values of the indicated mitosis markers in the five conditions are depicted as z-scores of normalized expression values (tpm) averaged over three replicates from the RNA-Seq data. (B) Fold changes of the indicated differentially expressed genes (top 25 up and downregulated after RCMV infection compared to mock-infected samples) of the four conditions indicated at the bottom compared to mock are shown as dotplots, with the size of the dot proportional to the –log10 transformed p-values, and the color indicative of the log2 transformed fold change. The genes are ordered according to an unsupervised clustering. [file Image_1.jpg]

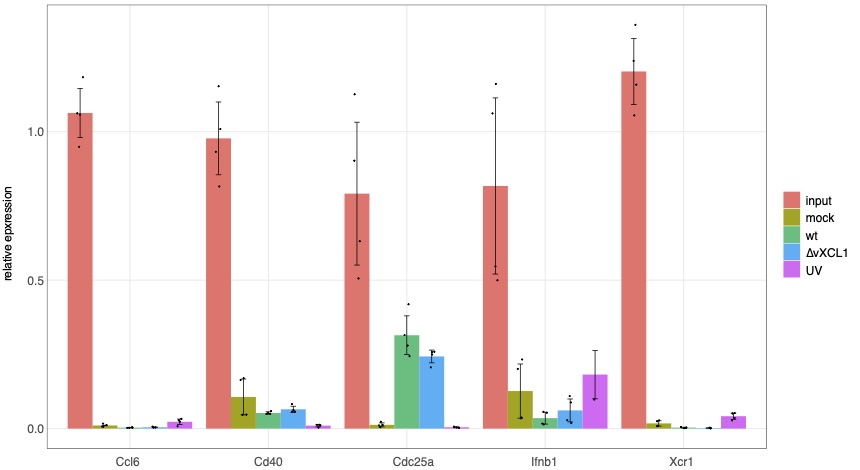

Supplement: Supplementary Figure 2 — Quantitative RT-PCR of mRNA extracted from OX-62-enriched DC from a total of two rats. After isolation and enrichment, DC were mock- or UV-infected or infected with wild-type or Δvxcl1 RCMV (see colored bars). RT-PCR was carried out to quantitate Ccl6, Cd40, Cdc25a, Ifnb1, and Xcr1 mRNA. Relative mRNA expression is shown on the ordinate. The housekeeping gene Ppia was used to normalize the data using the ΔΔ Ct method and input mRNA (red) was used as a reference to calculate ΔΔ Ct values. Averages and individual measurements of two rats each are shown. Error bars denote standard deviation. [file Image_2.jpg]
